# Global visual attention SPAN in different video game genres

**DOI:** 10.1038/s41598-023-49434-1

**Published:** 2023-12-11

**Authors:** Marc Argilés, Xavier González-Fortuny, Elisabet Fonts, Bernat Sunyer-Grau

**Affiliations:** 1 Departament d’Òptica i Optometria (DOO), Universitat Politècnica de Catalunya, BarcelonaTech (UPC), Campus de Terrassa, Edifici TR8, C.Violinista Vellsolà, 37, 08222 Terrassa, Barcelona, Spain; 2 School of Optics and Optometry, Violinista Vellsolà, 37, 08222 Terrassa, Catalonia Spain; 3 Centre de la Image i Tecnologia Multimèdia, Universitat Politècnica de Catalunya, Terrassa, Barcelona, Spain

**Keywords:** Psychology, Human behaviour

## Abstract

Video games, specifically action video games, have been demonstrated to be a useful tool in improving certain visual aspects in the general population. Visual attention span, the ability to simultaneously process multiple distinct visual elements during a single fixation, has been shown to improve among action video game players. The goal of this study was to verify that visual attention span is better not only in action video games, but also in other video game genres such as sports simulators or role-playing games. A total of 41 participants, aged 18 to 40 years old, were asked about the type of video games they were playing, name and genre, and the frequency of play. Visual attention span was assessed in all participants. Participants were divided into 4 groups according to the genre of video game they played. The total percentage of letter identification in the visual attention span was not significantly different between groups. A significant difference was found in the sixth position letter, and the right hemifield performance between groups, especially in sports simulators and action video game participants who showed a better performance. The action video game group showed a significant correlation between visual attention span performance and weekly hours played. Playing regularly different genres other than action video games can contribute to raise visual attention resources.

## Introduction

Widespread use of video games is very common in our world and includes a variety of platforms (television, smartphone, portable devices), which are used mainly for leisure and social time. According to Statista, video game players spend an average of 8.5 h a week playing video games^1^. Time play varies across countries but it confirms a global trend of video games becoming a new and popular form of entertainment. In 2023, there were approximately 3.07 billion active video gamers worldwide^2^. In parallel, electronic sports (eSports), competitions of video games regardless of the genre, are becoming increasingly popular with an audience of about 261.2 million people in 2022^3^.

Video games include a wide range of genres such as action, sports, multi-online battle arena (MOBA), role-playing games (RPG), music and social simulators. In particular, action video games (AVG) are the most studied genre in the research literature and have become an interesting tool due to their potential to improve certain visual functions^4–6^. These fast-paced commercial video games, for instance, Call of Duty®, Medal of Honor®, or Counter Strike®, create an environment to foster these visual skills, in contrast to other video game genres, more slow-paced, such as Tetris® or The Sims®^7^.

Some studies have shown an improvement in visual spatial attention due to AVG training such as useful field of view^5^, multi-object tracking^4^, attentional blink^5^, and in facilitating the learning process and modulating the resources to allocate attention more effectively during specific tasks^8,9^, for more information, see the reviews of Bavelier et al.^10^ and Bavelier and Green^11^. Because of these mechanisms, previous research has used AVG to improve vision abilities in a broad range of clinical applications such as visual acuity in amblyopia^12,13^, reading performance in dyslexia^14–16^, or improving visual and attention skills in brain damage^17,18^.

VA span refers to the capacity to process distinct visual elements simultaneously in one fixation and has been linked to reading performance^19–21^. In particular, AVG players have been shown to have a better performance in a visual attention task known as visual attention span (VA span)^22^. Antzaka et al.^22^ compared the performance between action and non-action video game players and concluded that VA span is better in action video game players, in line with previous studies about the possible effect of these fast-paced video games on the visual attention domain^23,24^.

However, other genres of video games than AVG, such as multi-battle online arenas (MOBA), role-playing games (RPG), and sports simulators (such as soccer, basketball or tennis), can include high visual attention demands that could also lead to higher visual processing. Despite that, to our knowledge, there is no research exploring the possible benefits of video game genres other than AVG in improving VA span. The purpose of our study was to replicate the experimental methodology of Antzaka et al.^22^, that is to evaluate va SPAN in AVG players and in non-video game players. In the present study, va SPAN has also been measured and compared in video game players of other genres such as MOBA/RPG and sports simulators. The author's hypothesis is that improvements in VA span are not limited to players of AVG but are extensive to players of other video game genres.

## Material and methods

### Recruiting and inclusion criteria

Participants aged between 18 and 40 years, all university students, were recruited from January to June 2023 from two different colleges in the Polytechnic University of Catalonia (Terrassa, Spain). All participants provided informed consent prior to enrolling in the study. The study protocol was approved by an institutional review board (11/2022, UPC) and conducted according to the tenets of the Declaration of Helsinki.

Participants were asked to fill out a questionnaire about the video games played and the amount of time spent with each one. The questionnaire consisted of a list of commercial video games and a checklist with three different weekly hours ranges: 0–1, 3–5, and 5–10 h/week. The participants were instructed to list the video games they had played in the past six months and to indicate the amount of time they had spent playing each game.

The video games selected were classified into three different genres: (a) action video games (AVG), (b) sports simulators (SIM), and (c) comprising both MOBA and RPG (MOBA/RPG). We provided commercial names in every genre to help participants select the time range. In order to be included in the study, participants had to play a minimum of 5 h/week in one category genre and no more than 1 h/week in the other genres. In addition, participants were asked to report as accurately as possible the number of hours per week spent playing video games, regardless of the genre, during the 6 months before the start of the study. Participants playing less than 1 h/week were included as non-video game players (NVG). Then, participants that meet the inclusion criteria were divided into 4 groups: (1) players of AVG, (2) players of sports simulators (SIM), (3) players of MOBA/RPG, and (4) non-video game players (NVG).

All participants had a best-corrected visual acuity of 20/20 in each eye at distance and near and a stereoacuity of 50" or better, evaluated with Randot Stereo Test (Stereo Optical Company, Chicago, USA). VA span was measured using the same methodology as Antzaka et al.^22^ using solely the global report, as the previously cited study did not find any advantage in the AVG group across the letter positions (i.e. partial report).

### Visual attention SPAN (va SPAN) assessment

**Figure 1:**
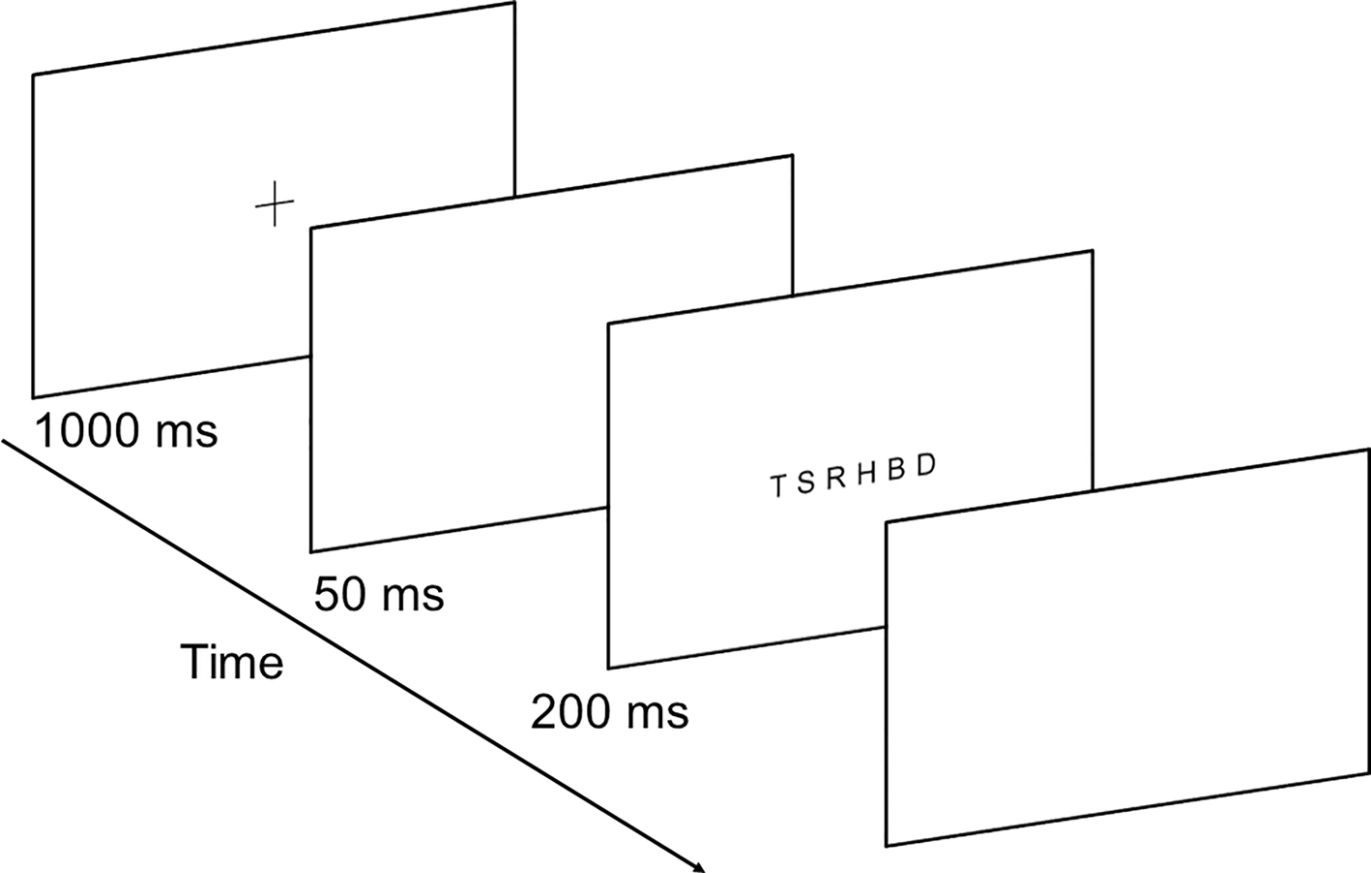
Schematic representation of the global VA span task.

The global report consisted of a sequence of six letters made out of 10 possible consonants (B, P, T, F, L, M, D, S, R, H), presented randomly in each trial and without letter repetition within the sequence. The whole string subtended a visual angle of 6º, using a laptop computer (Microsoft Surface Laptop 3, 2496 × 1664 pixels) at 60 cm of distance. Trials began with the presentation of a central fixation point for 1000 ms, followed by a blank screen for 50 ms, and displaying the string of letters for 200 ms (Fig. 1). Fixation point was presented between letters positions 3 and 4. Participants were verbally instructed to say the letters identified after each trial. Answers were recorded manually. A total of 24 trials were performed for each participant. Ten practice trials were done before the test to ensure that the task was completely understood.

Reading tasks were assessed in all participants in order to evaluate the reading process and its links with VA span performance. Similarly to the VA span task, we followed Antzaka et al.^22^ methodology for reading pseudowords. The reading task was subdivided into two tasks, PW1 and PW2. PW1 consisted of reading 21 words of 4 letters, with 2 additional letters added at the beginning of each word to mask sounding and thus creating pseudowords of 6 letters. PW2 was formed by words of 4 letters which incorporated 2 other letters in random positions, again making a pseudoword of 6 letters. Participants were asked to read the words out loud, written in Catalan language. Word presentation time and blank screens were the same as in the VA span task.

### Statistical analysis

VA span performance was computed as a percentage of accurately reported consonants for the total trials. A string of six letters was presented, each letter of the string was calculated as a percentage (correct identified letter/total presentations × 100). Global VA span was calculated as the median of all the percentages obtained from all the six-letter string presentations. Kolmogorov–Smirnov test was applied to check the normality of the data. Non parametric one-way ANOVA (Kruskal–Wallis test) was applied to study the differences of VA span between groups. The proportion of corrected letters identified in each hemifield was calculated to study the possible effect on their performance. The sum of percentages ([correct identified letter/total presentations] × 100) from letter positions 1 to 3 was considered as the global percentage for the left hemifield, and the sum of percentages from letter positions 3 to 6 as the right hemifield. Correlation analysis was calculated using Pearson (r) or Spearman (rho) test, depending on the parametric or non-parametric distribution from the Kolmogorov–Smirnov test. Mean comparison between groups was calculated using Mann–Whitney test (U test), in the case of non-normal distribution. Hemifield differences were compared between the sum of the correct percentage on the left, letters from 1 to 3, and on the right, letters from 4 to 6. Reading tasks were computed also as a correct percentage ([correct identified words/total presentations] × 100) for each task (PW1 and PW2). Chi-Square test (χ^2^) was used to study the possible difference between groups and genres. A *p*-value < 0.05 was used as a threshold of statistical significance. SPSS version 28 for Windows was used for the analysis. Sample size calculation was calculated using G*Power software^25^, and data from Antzaka et al.^22^. This free-availabe software includes a power analysis and is able to calculate a required sample size using input parameters (i.e. effect size and alfa error) and different power analyses depending on the statistical test (i.e. difference between two independent means or lineal bivariate regression). Moreover, G*Power can calculate post-hoc power analysis based on the results. For our study, we included the input using the same design and results from Antzaka et al., 2017, with a total of 42 participants, 13 per group, assuming an alpha risk of 0.05, 0.20 beta risk, a standard deviation of 5, and 7 for the minimum expected difference.

### Ethics approval

The study was conducted according to the guidelines of the Declaration of Helsinki and approved by the Institutional Review Board of the Universitat Politècnica de Catalunya (11/2022 UPC, 10 November 2022).

### Consent to participate

Informed consent was obtained from all individual participants included in the study.

## Results

A total of 41 participants were included in the study, 27 males and 17 females, ranging from 17 to 36 years, mean and standard deviation 21 (2.92) years old. Participants were distributed into the four groups according to their answers in the questionnaire: 13 (8 males, 5 females) were identified and included in the action video game group (AVG), 6 (4 males, 2 females) in the sports simulators (SIM) group, 13 (9 males, 4 females) in the multi-online battle arena and role-playing group (MOBA/RPG), and 9 (3 males, 6 females) in the non-video game players (NVG). Chi-Square test did not find any statistical differences between groups and genres (χ^2^ = 3.18,* p* = 0.365). Performance in both reading tasks measured as the percentage of correct words was not statistically significant between groups, PW1 (*p* = 0.319), and PW2, (*p* = 0.395).

Global percentage in the VA span, as the sum of all letter correct percentages, was not statistically significant between groups (*p* = 0.097). Mean and standard deviation in the AVG was 85.96 (9.10), SIM, 87.08 (7.61), MOBA/RPG, 79.55 (12.95), and NVG, 74.90 (9.86).

**Figure 2:**
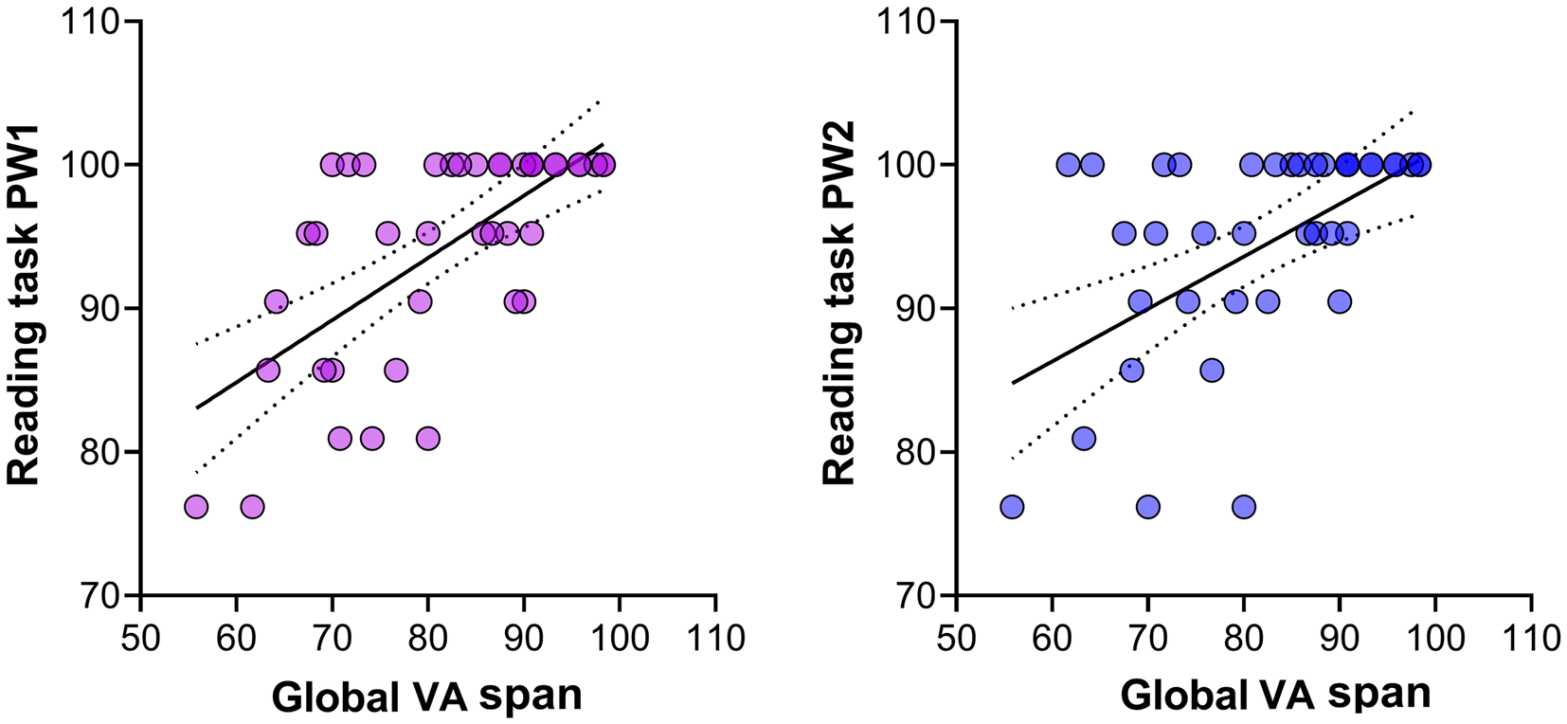
Correlation between global VA span (percentage of correctly identified letters of the six letters in the string) and reading task (PW1 left, PW2 right), as a percentage of correct identified words.

A moderate correlation was found between global percentage in the global VA span and reading pseudo-tasks, PW1 (rho(41) = 0.63, *p* < 0.0001), and PW2 (rho(41) = 0.52, *p* = 0.0004) (Fig. 2).

The VA span performance, depending on the position in the string (from 1 to 6), was compared between groups. No statistical differences were found in the first letter (Kruskal–Wallis test, (*p* = 0.193), second letter (*p* = 0.489), third letter (*p* = 0.697), fourth letter (*p* = 0.085), and fifth letter (*p* = 0.258).

A statistical difference was found in the sixth letter between groups (*p* = 0.008) (Table 1). Bonferroni post hoc multiple comparisons between groups found a statistical difference in the sixth letter between NVG and AVG, *p* = 0.005. No statistically significant differences were found in the 6^th^ letter between AVG and SIM, *p* > 0.90, nor between AVG and MOBA/RPG, *p* = 0.847, and NVG and SIM, *p* = 0.09.

**Table 1:** Descriptive results in each position of the letter identified in the global VA span report between groups.

Groups	Letter position in the VA span ( % correct letters)						Global VA span
	1	2	3	4	5	6	Total
AVG (n = 13)	100 (0.00)	89.61 (14.64)	86.92 (13.62)	79.23 (16.18)	78.84 (14.45)	81.18 (13.71)	85.96 (9.10)
SIM (n = 6)	98.33 (4.08)	95.83 (5.84)	86.67 (11.25)	72.50 (16.95)	84.16 (12.41)	85.00 (12.24)	87.08 (7.61)
MOBA/RPG (n = 13)	98.84 (2.19)	88.46 (10.48)	85.00 (15.27)	65.00 (20.41)	68.84 (21.90)	71.15 (21.61)	79.55 (12.95)
NVG (n = 9)	93.88 (13.17)	87.77 (19.22)	79.44 (17.93)	62.22 (12.27)	71.67 (15.20)	54.40 (15.89)	74.90 (9.86)
*p-value*	.193	.489	.697	.085	.258	.008	.097

Mean and standard deviation are shown in rows for each group.

*p*-values from ANOVA are shown in the last row.

**Figure 3:**
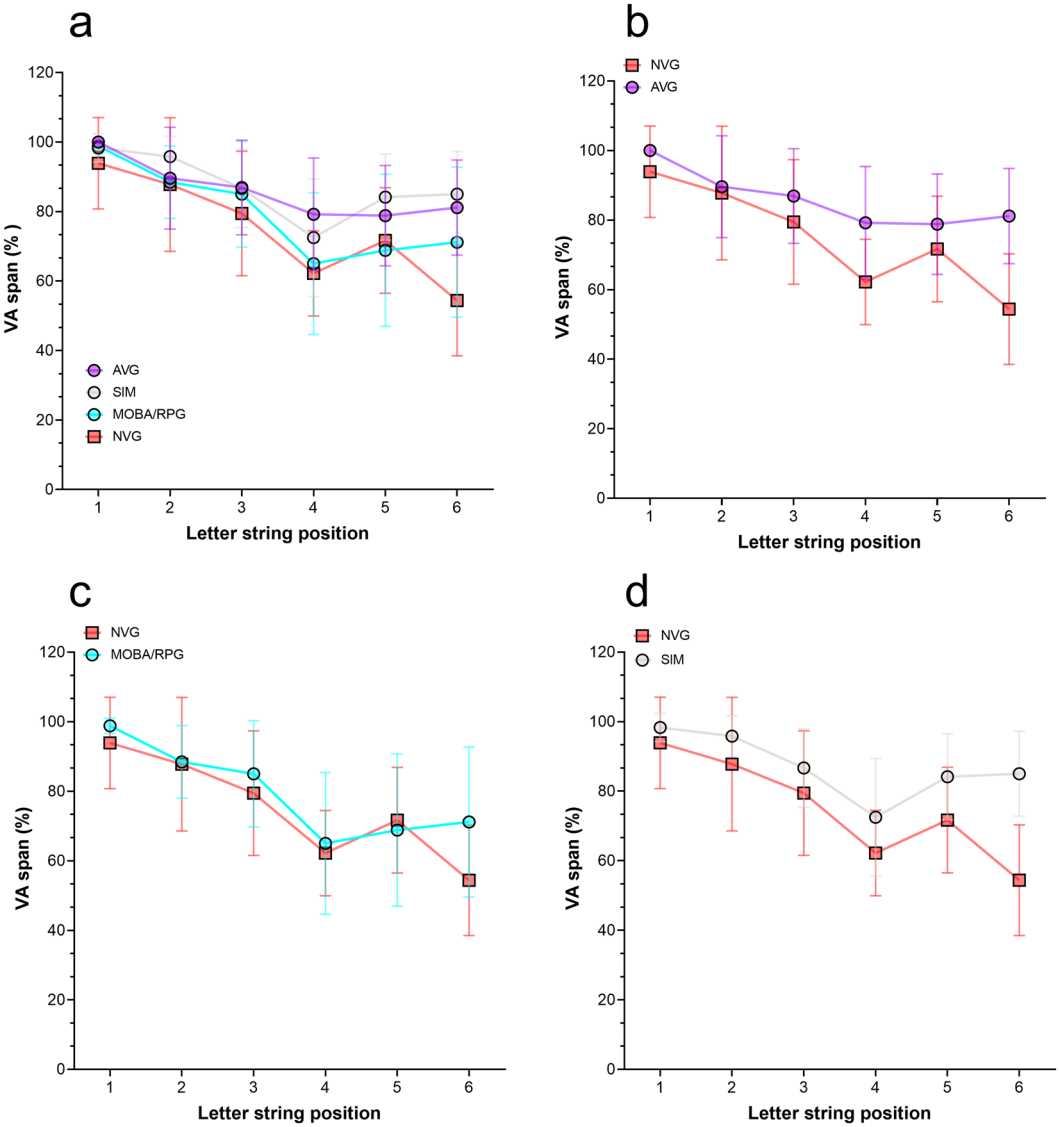
Differences in VA span performance for each letter position in the string (in percentage of correct letters) between groups. Mean and standard deviation are shown. (**a**): All groups, action video game group (AVG), sports simulators group (SIM), multi-online battle arena/role-playing group (MOBA/RPG), and non-video game (NVG), (**b**): NVG and AVG, (**c**): NVG and MOBA/RPG, (**d**): NVG and SIM.

Comparison between AVG and NVG revealed a statistically significant difference in overall VA span performance (U = 21.00, *p* = 0.011). Additionally, significant differences were observed in the identification of the fourth and sixth letters in the string (U = 23.00, *p* = 0.016) and (U = 10.50, *p* = 0.001), respectively. Figure 3 illustrates the VA span performance for each letter position across the four groups.

The correlation between hours played weekly and the percentage in VA span was computed in all groups. The correlation between all video game participants (n = 32) was not statistically significant (rho(32) = 0.22, *p* = 0.215). However, individual group analysis showed that only the AVG group (n = 13) showed a statistical correlation, *p* = 0.039, in contrast with SIM and MOBA/RPG, *p* = 0.419, *p* = 0.966, respectively.

**Figure 4:**
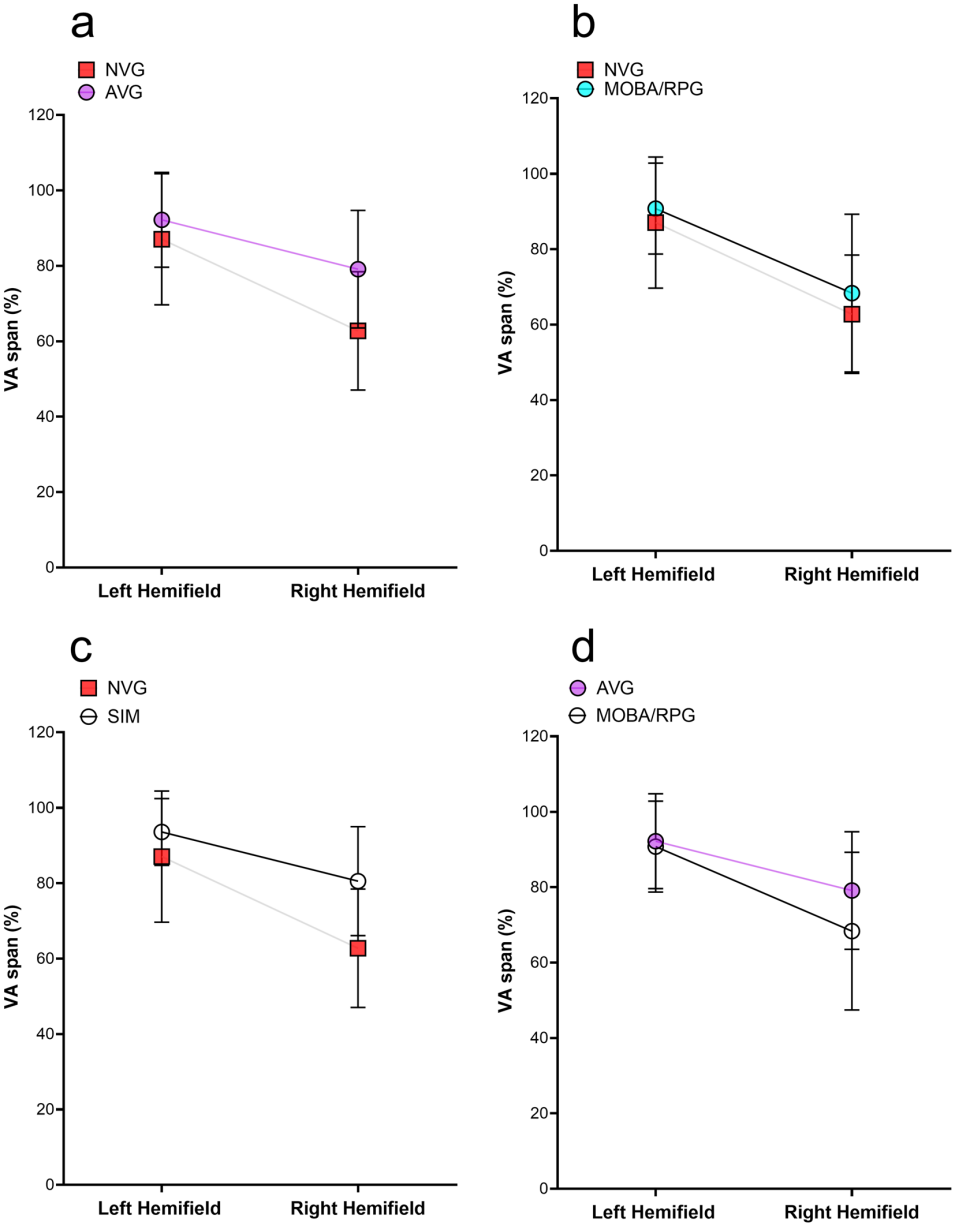
Differences in hemifield performance in the VA span task (in percentage of correct letters) between groups. Mean and standard deviation are shown. (**a**): non-video game (NVG) and action video game group (AVG), (**b**): non-video game (NVG) and multi-online battle arena/role-playing group (MOBA/RPG), (**c**): NVG and sports simulators group (SIM), and (**d**): AVG and MOBA/RPG.

Hemifield analysis from VA span performance showed no statistically significant differences in the left hemifield between groups, Kruskal–Wallis test (*p* = 0.435). However, right hemifield performance was statistically significant (*p* < 0.001) (Fig. 4). Multiple comparisons with Bonferroni correction found differences between AVG and MOBA/RPG, and between AVG and NVG, *p* = 0.043 and *p* = 0.002, respectively. The results in each hemifield and group are shown in Table 2. We found statistically significant differences between all participants (N = 41) and hemifields (*p* < 0.001), and between the AVG group, (*p* < 0.001), SIM (*p* = 0.005), MOBA/RPG, (*p* < 0.001), and NVG (*p* < 0.001).

**Table 2:** Descriptive results in each hemifield in the global VA span report between groups.

Groups	Hemifield analysis in the VA span	
	Left hemifield	Right hemifield
AVG (n = 13)	92.18 (12.60)	79.10 (15.59)
SIM (n = 6)	93.61 (8.87)	80.55 (14.44)
MOBA/RPG (n = 13)	90.77 (12.06)	68.33 (20.94)
NVG (n = 9)	87.04 (17.39)	62.77 (15.70)
*p -value*	.435	< .001

Mean and standard deviation are shown for both hemifields in all groups.

*p*-values from ANOVA are shown in the last row.

## Discussion

Our initial hypothesis was to investigate if other genres of video games, such as sports simulators or MOBA/RPG, can also be used for training studies to enhance visual spatial attention. To test this, we conducted a cross-sectional study comparing the performance in the global VA span in participants with high experience in each genre of videogame. For this study, a total of 41 participants, 27 males and 17 females, ranging from 17 to 36 years, were included and divided into four groups of study. VA span and two pseudo-reading tasks were conducted and compared between groups.

### VA span across videogame genre experience

In the present study, global VA span performance was not different between groups. However, the performance in identifying the letter shown at the right end (i.e. sixth and last letter), was significantly different, with better results in the action video game (AVG) and sports simulator (SIM) groups compared with the non-video game (NVG) group. This difference in the 6^th^ letter but not in the full sequence explains the better performance in the right hemifield, showing a left bias in video game participants compared with non-video games, especially in action video games and sports simulators. In addition, video game participants in all genres (AVG, SIM and MOBA/RPG) showed better performance in the VA span compared with non-video game players. Besides, if we compare only AVG and NVG we found a statistically significant difference in the global VA span performance, but not if we take into account the other video game genres. Antzaka et al.^22^ found better performance in the letter positions 4, 5 and 6 comparing action video game players and non-video game players, in contrast with our study which we found only in the 6^th^ letter position with all the groups.

In the hemifield analysis, we found better performance in the AVG, and also in the SIM group, compared with non-video game players. In our study, sports video game players performed better on the global VA span task, although not significant when compared to other groups, and showed better allocation of visual attention in the right hemifield than the AVG group. To our knowledge, there is no research explaining these results in players of sports simulation video games. However, existing published literature on AVG players shows different brain activation during verbal tasks^26^, including visual areas as in the occipital lobe^27^, and better functional connectivity between attentional and sensorimotor networks^28^. In addition, lower VA span performance, such as in developmental dyslexia, has been linked to reduced activation in the left superior parietal lobule^29^. Then, the asymmetries found in the global VA span between videogame genres, particularly SIM and AVG, seem to be related to different brain activation in visual attention tasks, especially during rapid visual simultaneous processing^30^. The better allocation of attentional resources in the right hemifield in videogame genres compared with non-videogame experience can also be indicative of better visuospatial resources in the periphery^4^. Hence, it seems that playing sports simulation video games can foster visual attention abilities with the same mechanisms that have been explored in AVG.

These results confirm our hypothesis that other genres such as sports simulators (i.e. soccer, tennis, basketball) can improve visual attentional abilities as well as AVG (i.e. first-person shooters). For instance, sports video games, such as soccer or tennis, create scenes where gamers need to be aware of the whole screen (i.e. periphery vision), and simultaneously pay attention to details in the centre (i.e. players and ball in movement), so that visual attentional resources are both distributed at the centre and the periphery as it happens with AVG video games. In MOBA/RPG video games, gamers need to be aware of the central screen with multiple items at the same time and also be aware of the minimap or other items outside the central field. Besides that, sports simulation video games seem to engage the visual attention resources better than MOBA/RPG, based on the global VA span and hemifield analysis.

### VA span and reading performance

Better performance in the global VA span has been linked to better reading abilities and attention resources^19–21^, which involves better activation of brain cortical networks such as the dorsal and ventral pathways^30^. Better performance in this task requires better attentional resources to process multi-parallel items, which can be explained by the fact that fast-paced video games, not only AVG, display specific characteristics that force video game players to foster their visual attention resources. We found similar results as Antzaka and colleagues (2017) regarding reading performance and VA span. A strong correlation was found in both pseudotasks and global VA span performance between all participants. Even though no statistical differences were found between groups in pseudo-reading tasks, we confirm the causal relationship between VA span and reading performance in our study. Hence, participants who had better global VA span read the pseudowords tasks more accurately, in accordance with previous literature^20,21^. VA span performance can be affected by multiple factors, such as reading experience^20^, or language^31^. In this study, all participants spoke the same languages (Spanish and Catalan), read both from left to right, and had no differences between reading pseudowords.

### VA span and learning

In the AVG group, a statistically significant correlation was found between hours played weekly and VA span performance. These results can be attributed to the fact that playing these video games engages learning resources and promotes brain plasticity through the visual system^32^. However, the number of hours played in video games of other genres such as sports simulators and MOBA/RPG were not statistically correlated with VA span performance. Some authors suggest that the possible transference in improvements in visual and auditory domains caused by playing action video games is time-dependent^33^. A possible explanation for these differences in correlation between time playing and VA span performance could be the differences in mechanisms that facilitate brain plasticity and transference in each domain for each video game genre.

### Limitations and future directions

Future training studies including action video games and sports simulators will confirm the causal relationship between VA span and playing specific video game genres. Our study has some limitations which make it difficult yet to establish this causal relationship. First, it comes from the participants sample, a post hoc analysis for the whole sample (N = 41) was calculated using G*Power software, for the 6-letter position using the one-way ANOVA fixed effect and alpha error 0.05. The power achieved was 0.97 (97%) for this study for global VA span analysis. However, the hemifield results gave a posthoc power of 0.66 (66%). Recruiting participants was especially difficult given the strict weekly hours for each group (> 5 h/week and < 1 h/week in the other genre) and took us 6 months of recruiting to complete our final sample of 41 participants.

Moreover, there is high variability in research using questionnaires to include participants in video game experience in cross-sectional studies. Some published literature consider the number of hours played in the last six months^34–36^, others in the last year^37^, and some in the past two years^38^, to consider the inclusion criteria for AVG and NAVG players. The threshold number of hours per week to classify participants between AVG and NAVG is also variable and ranges from a minimum of three hours per week to more than twenty hours per week^34–38^.There is also a freely available questionnaire developed from Bavelier Lab that can be used for recruiting participants into AVG and NAVG players, based on their videogame experience^39^. In our study, we use the same criteria for every group of video game experience. Future interventional studies training with different videogame genres will clarify the results obtained in our study. However, we consider that our results using a strong criteria of inclusion between groups postulate that not only AVG can foster visual attention abilities as previous research had shown, but sports videogames such as football or tennis or MOBA/RPG can also be considered.

Gender bias in our study was an additional factor, even though other studies have been published with such bias^40,41^. In the present study, of the 41 participants, 17 were females, and Chi-Square test did not find any significant difference between groups. For instance, in Antzaka et al.^22^ action video games group of 19 participants, 14 were males. In the AVG group of the present study, five of the 13 participants were women, in the sports simulation group 2 out of 6 were women, 4 females in the MOBA/RPG and 6 in the NVG. Other cross-sectional research studies involving recruiting participants in action and non-action video game groups have been published with gender bias, which could be explained by the fact that males generally play more video games than females^42^.

### Conclusions

Visual attention distribution and allocation of resources during the VA span task are better in sports simulation and in action video game players, especially in the right hemifield, than in MOBA/RPG and non-video game players. Future work can also contemplate other genres of video games to study the possible applications to improve visual functions.

## Data Availability

Data is available at the following OSF link: https://osf.io/za647/?view_only=f71cc6a2cda143e4be8df484814a0ee2. Any question should be addressed to Marc Argilés (marc.argiles@upc.edu).
